# Revisit of an unanswered question by pooled analysis of eight cohort studies in Japan: Does cigarette smoking and alcohol drinking have interaction for the risk of esophageal cancer?

**DOI:** 10.1002/cam4.2514

**Published:** 2019-09-01

**Authors:** Isao Oze, Hadrien Charvat, Keitaro Matsuo, Hidemi Ito, Akiko Tamakoshi, Chisato Nagata, Keiko Wada, Yumi Sugawara, Norie Sawada, Taiki Yamaji, Mariko Naito, Keitaro Tanaka, Taichi Shimazu, Tetsuya Mizoue, Shoichiro Tsugane, Manami Inoue

**Affiliations:** ^1^ Division of Cancer Epidemiology and Prevention Department of Preventive Medicine Aichi Cancer Center Research Institute Nagoya Japan; ^2^ Epidemiology and Prevention Group Research Center for Public Health Sciences National Cancer Center Tokyo Japan; ^3^ Division of Cancer Information and Control Department of Preventive Medicine Aichi Cancer Center Research Institute Nagoya Japan; ^4^ Department of Epidemiology Nagoya University Graduate School of Medicine Nagoya Japan; ^5^ Department of Public Health Hokkaido University Graduate School of Medicine Sapporo Japan; ^6^ Department of Epidemiology and Preventive Medicine Gifu University Graduate School of Medicine Gifu Japan; ^7^ Division of Epidemiology, Department of Public Health and Forensic Medicine Tohoku University Graduate School of Medicine Sendai Japan; ^8^ Department of Oral Epidemiology Hiroshima University Graduate School of Biomedical and Health Sciences Hiroshima Japan; ^9^ Department of Preventive Medicine, Saga Medical School, Faculty of Medicine Saga University Saga Japan; ^10^ Department of Epidemiology and Prevention Center for Clinical Sciences National Center for Global Health and Medicine Tokyo Japan

**Keywords:** alcohol drinking, cigarette smoking, esophageal cancer, interaction, pooled analysis

## Abstract

Cigarette smoking and alcohol drinking are two major risk factors for esophageal cancer. Not all, but several of case‐control studies have indicated interaction between the two factors; however, no prospective study has validated this phenomenon to date. Therefore, the interaction between smoking and alcohol drinking is still open‐ended question. To answer this, we conducted a pooled analysis using large‐scale population‐based cohort studies in Japan. Male subjects from eight cohort studies were included. Cigarette smoking and alcohol drinking were both categorized categorically (never/ever), and in the three consumption levels of pack years and ethanol consumption/day. Effects of smoking and drinking in each study were estimated by Cox regression models. The study‐specific results were combined through meta‐analysis to obtain summary effects of hazard ratios (HRs) and measures of interactions at both additive and multiplicative scales. Population attributable fractions (PAFs) from smoking and drinking were obtained using distributions of exposures and fully adjusted HRs. In 162 826 male subjects, 954 esophageal cancer incidences were identified. HRs of ever smoking, ever drinking, and their combination were 2.92 (1.59‐5.36), 2.73 (1.78‐4.18), and 8.86 (4.82‐16.30), respectively. Interaction between cigarette smoking and alcohol drinking was significantly positive on the additive scale, but not significant on the multiplicative scale. The joint effect of smoking and drinking in three levels of evaluation showed a similar significant super‐additive interaction. PAFs from smoking, drinking, and their combination were 55.4%, 61.2%, and 81.4%, respectively. Cigarette smoking and alcohol drinking had a significant positive additive interaction for esophageal cancer risk.

## Graphical abstract

A significant positive additive interaction between cigarette smoking and alcohol drinking for the risk of esophageal cancer was found, although significant interaction on a multiplicative scale was not observed. Population attributable fraction suggest that either quitting smoking or drinking alone might make a major contribution to esophageal cancer prevention.

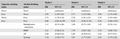

## 1 INTRODUCTION

Cigarette smoking and alcohol drinking are serious public health issues. WHO has estimated that 8.7% of global deaths in 2004 and 3.7% of disability‐adjusted life years (DALYs) were attributable to cigarette smoking.^1^ Moreover, alcohol drinking caused 3.6% of deaths and 4.4% of DALYs. An accurate understanding of the risk of cigarette‐ and alcohol‐related diseases in each population will allow the current impact of these practices to be identified. In addition, this understanding of risk will also be useful in predicting the effect of intervention, because cigarette smoking and alcohol drinking are modifiable risk factors.

Both cigarette smoking and alcohol drinking have been established as major risk factors for esophageal cancer.^2^, ^3^, ^4^, ^5^, ^6^ Biological evidence supports the idea that tobacco extracts and ethanol have an interactive effect in carcinogenesis.^7^, ^8^ To support this, several, but not all, case‐control studies showed that cigarette smoking and alcohol drinking have an interactive effect on esophageal cancer risk.^9^, ^10^, ^11^, ^12^, ^13^, ^14^, ^15^, ^16^, ^17^, ^18^, ^19^, ^20^, ^21^, ^22^, ^23^, ^24^, ^25^ In contrast, four cohort studies and a case‐cohort study have failed to replicate this interactive effect of two factors 26, 27, 28, 29, 30, 31 (Table 1). Therefore, although the interaction between smoking and alcohol drinking has been recognized as an answered question,^32^ this question remained an unanswered one warranting a further investigation in prospective cohort studies.

**Table 1:** Studies reporting joint effect of smoking and alcohol for esophageal cancer

Study	Ref	Journal	Year	Country	Study Design	Category of smoking	Category of drinking	RR of heaviest smoking and CI	RR of heaviest drinking and CI	Joint effect of heaviest smoking and drinking			Statistical test of multiplicative interaction	Statistical test of additive interaction
Kinjo et al.	[26]	J Epidemiol	1998	Japan	Cohort	Never/Current	Non‐daily/daily	1.6 (1.1‐2.1)	1.0 (0.4‐2.0)	3.9 (2.7‐5.4)			NE	NE
Fan et al.	[27]	Nutr Cancer	2000	China	Cohort	Number of years of smoking	Number of drinks per day	2.18 (0.80‐5.92)	3.86 (0.86‐17.26)	8.00 (3.36‐19.05)			*P* = .99	NE
Ishiguro et al.	[28]	Cancer lett	2009	Japan	Cohort	Light/heavy	Light/heavy	2.31 (1.58‐3.38)	2.96 (2.01‐4.34)	6.12 (4.13‐9.05)			*P* = .70	NE
Weikert et al.	[29]	Int J Cancer	2009	Europe	Cohort	Never/ex/current	Lifetime alcohol intake	5.76 (3.20‐10.36)	1.71 (0.38‐7.67)	22.86 (12.27‐42.60)			*P* = .27	NE
Yaegashi et al.	[30]	Asian Pac J Cancer Prev	2014	Japan	Cohort	Smoking status	Drinking status	0.80 (0.15‐4.15)	0.64 (0.07‐5.96)	6.05 (1.87‐19.60)			NE	NE
Steevens et al.	[31]	Gut	2010	Nether‐lands	Case‐cohort	never/former/current	gram ethanol/day	1.70 (0.72‐4.05)	3.74 (1.25‐11.20)	8.05 (3.89‐16.60)			*P* = .65	NE
De Stefani et al.	[9]	Cancer Res	1990	Uruguay	Case‐control	Cigarettes per day	ml per day	3.10	6.70	22.60			NE	NE
Chongsuvivatwong	[10]	J Gastroenterol Hepatol	1990	Thailand	Case‐control	Never/ever	Never/Ever	1.65 (0.66‐4.11)	4.73 (0.53‐42.1)	5.68 (2.14‐15.1)			NE	NE
Brown et al.	[11]	J Natl Cancer Inst	1994	America	Case‐control	Light/heavy	Drinks per week	4.5 (1.4‐14.6)	31.0 (9.8‐98.5)	149.2 (39.2‐567.4)			Described as “not significantly different” in text	Described as “statistically different” in text
Castellsague et al.	[12]	Int J Cancer	1999	South America	Case‐control	Average number of cigarettes smoked per day	Average amount of pure ethanol consumed per day	6.84	14.13	50.85			*P* = .20	NE
Zambon et al.	[13]	Int J Cancer	2000	Italy	Case‐control	Smoking habit (cig/day)	Alcohol intake (drinks/week)	6.97 (3.22‐15.06)	28.48 (10.09‐80.39)	130.32 (15.20‐980.10)			*P* = .27	NE
Znaor et al.	[14]	Int J Cancer	2003	India	Case‐control	Never/Ever	Never/Ever	3.57 (2.51‐5.06)	3.41 (1.46‐7.99)	7.33 (5.06‐10.62)			*P* = .62	NE
Lee et al.	[15]	Int J Cancer	2005	Taiwan	Case‐control	never/ever	never/ever	2.4 (1.1‐5.0)	1.4 (0.4‐4.5)	23.3 (12.2‐44.5)			*P* = .003	NE
Wu et al.	[16]	Eur J Clin Invest	2006	China	Case‐control	Never/Ever	Never/Ever	6.5 (1.9‐29.80)	23.3 (4.3‐142.2)	108.0 (35.1‐478.0)				NE
Hashibe et al.	[17]	Int J Cancer	2007	Central and Eastern Europe	Case‐control	No/Yes	No/Yes	0.71 (0.07‐7.00)	0.96 (0.28‐3.28)	6.42 (2.03‐20.30)			9.41 (0.88‐100.27)*P * < 0.01	NE
Lee et al.	[18]	Int J Cancer	2007	Taiwan	Case‐control	never/ever	never/ever	1.9 (1.2‐3.2)	2.3 (1.2‐4.4)	19.7 (12.4‐31.3)			*P* = .0001	8.2 (4.1‐16.5)
Lee et al.	[19]	Int J Cancer	2008	Taiwan	Case‐control	No/Yes	No/Yes	1.2 (0.2‐7.7)	3.7 (0.5‐27.1)	45.0 (12.0‐168.3)			*P* = .042	NE
Tanaka et al.	[20]	Gut	2010	Japan	Case‐control	never/ever	never/ever	4.5 (1.3‐15.9)	1.5 (0.7‐3.3)	5.0 (2.5‐10.1)			*P* <.001	NE
Canova et al.	[21]	Tumori	2010	Italy	Case‐control	Pack year	average drinks/day	3.36 (1.39‐8.15)	1.65 (0.60‐4.56)	34.81 (14.69‐82.50)			*P* = .0012	NE
Chen et al.	[22]	Exp Ther Med	2010	China	Case‐control	Never/Ever	Never/Ever	6.2	19.5	43.8			beta = ‐1.01 (‐1.46 ‐ −0.56)	NE
Wu et al.	[23]	Cancer Causes Control	2011	China	Case‐control	Never/Ever	Never/Ever	1.20 (0.96‐1.51)	1.03 (0.80‐1.32)	2.10 (1.72‐2.56)			*P* <.001	NE
Anantharaman et al.	[24]	Oral Oncol	2011	Europe	Case‐control	Never/Ever	Never/Ever	2.68 (1.69‐4.24)	3.14 (1.50‐6.55)	7.86 (4.81‐12.86)			0.93 (0.42‐2.03)	NE
Lin et al.	[25]	Int J Cancer	2015	China	Case‐control	Never/Ever	Never/Ever	2.26 (1.31‐3.92)	1.28 (8.00‐2.04)	5.58 (3.88‐8.03)			NE	NE

Abbreviations: Ref, reference number; RR, relative risk; CI, confidence interval; NE, not estimated

There are two important points in evaluating the interaction between cigarette smoking and alcohol drinking for esophageal cancer risk in cohort studies. First, only two studies reported the magnitude of interaction for esophageal cancer risk.^15^, ^17^ However, this information is necessary if the attribution of cigarette smoking and alcohol drinking is to be estimated. Greater statistical power is required to assess the relevant magnitude of interaction on esophageal cancer risk. Second, interaction between two exposures should be assessed both multiplicatively and additively. Previous evaluations often measured the interaction on a multiplicative scale, because this measure could be obtained calculated by most software packages. In contrast, interaction was rarely measured on an additive scale because of the additional work required to estimate measures of additive interaction.^15^, ^33^, ^34^ Nevertheless, additive interaction is a relevant measure for assessing the public health significance of interaction, because tests for additive interaction sometimes have greater power to detect an interaction than tests for multiplicative interaction.^34^ In addition, interaction on an additive scale might be more appropriate than multiplicative interaction when estimating the impact of interventions.^35^, ^36^ Furthermore, type of interaction might suggest theory of carcinogenesis. Under a multistage theory of carcinogenesis, additivity of action of two factors might occur if each act independently on the same stage, whereas multiplicativity of action might be observed if each factor acts on a different carcinogenic stage.^37^ In this context, additive and multiplicative interactions represent a way of quantifying departure from these two hypotheses about the effect of the factors on the carcinogenic process. Accordingly, interaction between cigarette smoking and alcohol drinking for esophageal cancer risk should be assessed quantitatively on both an additive and multiplicative scale.

Here, we conducted a pooled analysis using large‐scale population‐based cohort studies in Japan to accurately estimate the joint effect of cigarette smoking and alcohol drinking on esophageal cancer risk.

## 2 METHOD

### 2.1 Study population

In 2006, the Research Group for the Development and Evaluation of Cancer Prevention Strategies in Japan began pooling original data from major cohort studies to evaluate the association between lifestyle and major cancers in Japanese, along with systematic reviews of the relevant literature. Topics for the pooled analysis were determined based on their scientific and public health importance, as determined by discussions among group members. For the present analysis, the following a priori inclusion criteria were established: population‐based cohort studies conducted in Japan; study initiation between the mid‐1980s and mid‐1990s; inclusion of more than 30 000 participants; and baseline collection of information on diet, alcohol intake, and smoking.

Subjects in this study were from eight representative large‐scale cohort studies in Japan: (a) the Japan Public Health Center‐based Prospective Study (JPHC‐I),^38^ (b) JPHC‐II,^38^ (c) the Japan Collaborative Cohort Study (JACC),^39^ (d) the Ohsaki National Health Insurance Cohort Study (OHSAKI),^40^ (e) the Miyagi Cohort Study (MIYAGI),^41^ (f) the Three‐Prefecture Cohort Study, Miyagi (3‐pref MIYAGI),^42^ (g) the Three‐Prefecture Cohort Study, Aichi (3‐pref AICHI),^42^ and (h) the Takayama Study (TAKAYAMA)^43^ (Table 1). These studies all commenced after the mid‐1980s and each enrolled more than 30 000 participants. Residence status in each study, including survival, was confirmed through the residential registry. Female subjects were excluded because the numbers of incident esophageal cancer cases, cigarette smokers, and alcohol drinkers were too small to analyze. Variables used in data linkage, censoring criteria, and the method used to obtain information on cancer incidence in each study are provided in Table S1. Study quality was assessed using Newcastle‐Ottawa Scale. The scores were eight in all the cohort studies.^44^ Each study was approved by its relevant institutional ethics review board.

### 2.2 Assessment of exposure

Exposure data were retrieved from self‐administered questionnaire surveys conducted at baseline in each study. For cigarette smoking status, subjects were categorized as never or ever smokers. Cumulative cigarette smoking was evaluated using pack‐years, calculated by multiplying the number of packs consumed per day by the number of years of smoking, then classified into the three categories of 0, 0< and ≤40, and >40 pack‐years. For alcohol drinking status, subjects were categorized as never or ever drinkers. Alcohol drinking was categorized by amount as <23, 23 to <46, or ≥46 ethanol g/day. The cut point of 23 g ethanol was defined on the basis that some questionnaires collected consumption data in terms of one “go” (180 mL), a term for a Japanese sake equivalent, which contains 23 g ethanol.

### 2.3 Assessment of outcome

The individual studies identified esophageal cancer cases via local cancer registries or direct access to major local hospitals. Information on cancer diagnosis was collected for the whole population and was coded using the International Classification of Diseases for Oncology, Third Edition (ICD‐O3).^45^ Study outcome was defined as the incidence of esophageal cancer (ICD‐9:150.0‐150.9,^46^ ICD‐10^46^ or ICD‐O3: C15.0‐C15.9) during the follow‐up period of each study. Participants were followed from the date of completion of the baseline questionnaire (JPHC‐I and ‐II: 1990‐1994, JACC: 1998‐1990, MIYAGI: 1990, 3‐pref. MIYAGI: 1984, OHSAKI: 1994, 3‐pref. AICHI: 1985, and TAKAYAMA: 1992), date of death, date of loss to follow‐up, or date of diagnosis of esophageal cancer, whichever occurred first.

### 2.4 Statistical analysis

The analysis was based on a three‐step approach: first, Cox regression models were used in each study to obtain estimates of the effect of smoking and drinking; second, these study‐specific results were combined through meta‐analysis; and finally, the summary effects were used to compute various measures of interaction.

### 2.5 Estimation of the study‐specific effects of smoking and alcohol consumption

In the context of categorical variables (here, smoking and alcohol consumption), the calculation of interaction measures relies on fitting models saturated for the relationship between these factors (ie, we estimated a different hazard ratio for each combination of categories). Potential confounders were considered to be age at baseline (continuous), study area (only for JPHC‐I, ‐II, and JACC), body mass index (BMI, 14 to <19, 19 to <21, 21 to <23, 23 to <25, 25 to <27, 27 to <30 kg/m^2^, and missing), and vegetable and fruit intake (never, 1‐2 days/week, 3‐4 days/week, 5‐7 days/week, and missing). The study estimated two types of hazard ratios (HRs): Model 1, which adjusted for age and area; and Model 2, which adjusted for age, area, BMI, and vegetable and fruit intake. We further estimated HRs which excluded cases within two years of baseline (Model 3).

### 2.6 Estimation of summary effects by meta‐analysis

Because the effects of smoking and alcohol in the previously described multivariable Cox models were estimated jointly, we used multivariate meta‐analysis^47^, ^48^ to account for the fact that estimates coming from the same study were correlated. More precisely, we conducted random effects multivariate analyses based on likelihood maximization.^47^ Firth correction was used to deal with perfect prediction, that is, the fact that there might be no cases for some combinations of risk factor categories.

### 2.7 Measures of interaction

The interaction of two risk factors refers to the fact that the effect of exposure to one factor might be modified by exposure to the second factor. In accordance with current interpretation of what a *modification* of the effect represents (which might depend on available knowledge on the biological mechanisms underlying the effects of the factors), two main types of interaction can be defined, an additive interaction and a multiplicative interaction. To explain these concepts, consider two binary risk factors, E1 and E2, and RR_10_, RR_01_, and RR_11_, the relative risks corresponding respectively to exposure to E1 only, E2 only, and to the combined exposure to E1 and E2 (note that we can extend this notation with RR_00_ = 1 corresponding to the relative risk for the nonexposed). Now say that RR_10_ = 2 and RR_01_ = 3. The additivity of the effects of the factors would correspond to the situation where RR_11_ = 4, that is, RR_11_ − RR_10_ = RR_01_ − 1, whereas multiplicativity would correspond to the situation where RR_11_ = 6, that is, RR_11_ = RR_10_ × RR_01_. According to the theory of how factors interact (at the biological level) to increase the risk of disease occurrence, we might understand “effect measure modification” as expressing a departure from multiplicativity (ie, RR_11_ ≠ RR_10_ × RR_01_) or, and more commonly in the epidemiological context, as expressing a departure from additivity (ie, RR_11_ − RR_10_ ≠ RR_01_ − 1).

From a computational point of view, multiplicative interaction can usually be assessed very easily because commonly used models (logistic regression, Poisson regression, Cox model) specify a multiplicative relationship between the factors. Consequently, multiplicative interaction can be assessed through the *interaction term* included between the factors in the formula of the model. In the case of additive interaction, we calculated in this work three commonly used measures, namely the relative excess risk due to interaction, RERI = RR_11_ − RR_10_ − RR_01_ + 1; the synergy index, Syn = (RR_11_ − 1)/[(RR_10_ − 1) + (RR_01_ − 1)]; and the attributable proportion, AP = (RR_11_ − RR_10_ − RR_01_ + 1)/RR_11_.^49^ RERI > 0, Syn > 1, and AP > 0 means positive (super‐additive) interaction.

In the case of categorical variables with more than two categories, we considered category by category interactions; that is, if E1 had N1 categories and E2 had N2 categories, the interaction measures between exposure to category $i\;(2\leq i\leq N_{1})$ of E1 and to category $j\;(2\leq j\leq N_{2})$ of E2 were based on the preceding formulas, replacing ${\text{RR}}_{10}$ by ${\text{RR}}_{i1}$, ${\text{RR}}_{01}$ by ${\text{RR}}_{1j}$, ${\text{RR}}_{11}$and by ${\text{RR}}_{\mathrm{ij}}$.

### 2.8 Confidence interval estimation for the summary estimates

For the summary (meta‐analytic) estimates of hazard ratios and multiplicative interactions, confidence intervals (CIs) were obtained by simple computation on the basis that these quantities were monotonous transformations of linear combinations of the estimated summary coefficients.

For the summary measures of additive interactions, CIs were obtained by Monte Carlo simulation (sample n = 1 000 000) in the multivariate distribution of summary coefficients, assuming that this distribution is a multivariate normal.

### 2.9 Population attributable fractions

Population attributable fractions (PAFs) were obtained by using the distribution of exposure to smoking and drinking in the study population and the summary hazard ratios of the fully adjusted model (Model 2).

PAFs for two the dichotomous exposures (E1, E2) were calculated by the following formulas:

$$\begin{array}{c} \text{PAF}\left(E1\right):\\ \;\frac{p_{10}*\left({\text{RR}}_{10}-1\right)+p_{11}*\left({\text{RR}}_{11}-{\text{RR}}_{01}\right)}{1+\left[p_{10}*\left({\text{RR}}_{10}-1\right)+p_{01}*\left({\text{RR}}_{01}-1\right)+p_{11}*\left({\text{RR}}_{11}-1\right)\right]} \end{array}$$

$$\begin{array}{c} \text{PAF}\left(E2\right):\\ \;\frac{p_{01}*\left({\text{RR}}_{01}-1\right)+p_{11}*\left({\text{RR}}_{11}-{\text{RR}}_{10}\right)}{1+\left[p_{10}*\left({\text{RR}}_{10}-1\right)+p_{01}*\left({\text{RR}}_{01}-1\right)+p_{11}*\left({\text{RR}}_{11}-1\right)\right]} \end{array}$$

$$\begin{array}{c} \text{PAF}\left(E1+E2\right):\\ \;\frac{p_{10}*\left({\text{RR}}_{10}-1\right)+p_{01}*\left({\text{RR}}_{01}-1\right)+p_{11}*\left({\text{RR}}_{11}-1\right)}{1+\left[p_{10}*\left({\text{RR}}_{10}-1\right)+p_{01}*\left({\text{RR}}_{01}-1\right)+p_{11}*\left({\text{RR}}_{11}-1\right)\right]} \end{array}$$

Similarly, PAFs for the two categorical exposures with three levels each (numbered 1 to 3) were calculated by the following formulas:

$$\text{PAF}\left(E1\right):\frac{\sum_{i=1}^{3}\sum_{j=1}^{3}p_{\mathrm{ij}}\left({\text{RR}}_{\mathrm{ij}}-{\text{RR}}_{1j}\right)}{1+\sum_{i=1}^{3}\sum_{j=1}^{3}p_{\mathrm{ij}}\left({\text{RR}}_{\mathrm{ij}}-{\text{RR}}_{11}\right)}$$

$$\text{PAF}\left(E2\right):\frac{\sum_{i=1}^{3}\sum_{j=1}^{3}p_{\mathrm{ij}}\left({\text{RR}}_{\mathrm{ij}}-{\text{RR}}_{i1}\right)}{1+\sum_{i=1}^{3}\sum_{j=1}^{3}p_{\mathrm{ij}}\left({\text{RR}}_{\mathrm{ij}}-{\text{RR}}_{11}\right)}$$

$$\text{PAF}\left(\text{E1}+\text{E2}\right):\frac{\sum_{i=1}^{3}\sum_{j=1}^{3}p_{\mathrm{ij}}\left(RR_{\mathrm{ij}}-RR_{11}\right)}{1+\sum_{i=1}^{3}\sum_{j=1}^{3}p_{\mathrm{ij}}\left(RR_{\mathrm{ij}}-RR_{11}\right)}$$

where RR_11 = 1 is the relative risk in the reference category of exposure to both factors.

## 3 RESULTS

The present pooled analysis included eight cohort studies, comprising 162 826 male subjects with 954 incident esophageal cancer cases during 2 053 871 person‐years of follow‐up (average follow‐up 12.6 years) (Table 2). At baseline, the proportion of ever smokers and ever drinkers was 60.6% and 78.5%, respectively. Almost half of subjects (49.1%) experienced both cigarette smoking and alcohol drinking. Cumulative smoking and amount of alcohol drinking were evaluated in five cohort studies (JPHC‐II, JACC, MIYAGI, OHSAKI, and TAKAYAMA). The proportion of subjects with heaviest smoking and drinking (more than 40 pack‐years of smoking and consumption of 46 g or more ethanol a day) was 8.1% (Table S2).

**Table 2:** Characteristics

Characteristics of the cohort studies									Characteristics of subjects in the present study			
Study	Population	Age (years) at baseline survey	Year(s) of baseline survey	Population size	Rate of response (%) to baseline questionnaire	Method of follow‐up	Last follow‐up time	Mean duration of follow‐up (years)	Age range (years)	Mean duration of follow‐up (years)	Size of cohort	Number of esophageal cancer incidence
JPHC‐I	Japanese residents of 5 public health center areas in Japan	40‐59	1990	61 595	82%	Cancer registries and death certificates	2009	17.5	40‐59	17.3	20 258	144
JPHC‐II	Japanese residents of 6 public health center areas in Japan	40‐69	1993‐1994	78 825	80%	Cancer registries and death certificates	2007	13.0	40‐69	12.5	29 188	181
JACC	Residents from 45 areas throughout Japan	40‐79	1988‐1990	110 585	83%	Cancer registries (selected areas: 24) and death certificates	2009	13.0	40‐79	13.1	25 547	128
MIYAGI	Residents of 14 municipalities in Miyagi Prefecture, Japan	40‐64	1990	47 605	92%	Cancer registries and death certificates	2007	15.6	40‐64	15.3	22 908	167
OHSAKI	Beneficiaries of National Health Insurance among residents of 14 municipalities in Miyagi Prefecture, Japan	40‐79	1994	54 996	95%	Cancer registries and death certificates	2006	8.9	40‐79	8.8	22 516	154
3‐pref MIYAGI	Residents of 3 municipalities in Miyagi Prefecture, Japan	40‐98	1984	31 345	94%	Cancer registries and death certificates	1992	7.4	40‐98	7.3	12 400	54
3‐pref AICHI	Residents of 2 municipalities in Aichi Prefecture, Japan	40‐103	1985	33 529	90%	Cancer registries and death certificates	2000	11.5	40‐97	11.2	15 582	59
TAKAYAMA	Residents of Takayama city in Gifu Prefecture, Japan	35‐	1992	31 552	85%	Cancer registry and death certificate	2008	13.6	35‐98	13.2	14 427	67
Total				450 032						12.6	162,826	954

Abbreviations: JACC, The Japan Collaborative Cohort Study; JPHC, Japan Public Health Center‐based Prospective Study; MIYAGI, The Miyagi Cohort Study; OHSAKI, Ohsaki Cohort Study; 3‐pref MIYAGI, The Three Prefecture Cohort Miyagi; 3‐pref AICHI, The Three Prefecture Cohort Aichi; TAKAYAMA, The Takayama Study.

Esophageal cancer risk of smoking status, drinking status, and their interactions are shown in Table 3. In the age‐ and area‐adjusted model (model 1), HRs and their 95% CIs of cigarette smoking, alcohol drinking, and their combination were 2.92 (1.59‐5.36), 2.73 (1.78‐4.18), and 8.86 (4.82‐16.30), respectively. A statistically significant and positive interaction in the additive scale was observed with an RERI of 4.21 (2.26‐8.13), synergy of 2.16 (1.83‐2.77), and AP of 0.48 (0.40‐0.54). When the interaction was evaluated multiplicatively, the interaction of smoking and alcohol was 1.11 (0.74‐1.69). The direction of multiplicative interaction was the same as that for additive interaction, but the magnitude was small and without statistical significance. The multivariate‐adjusted HRs and HRs with the exclusion of early cases were similar to those in model 1.

**Table 3:** Cigarette smoking status, alcohol drinking status, and their interaction for the risk of esophageal cancer

Cigarette smoking status	Alcohol drinking status	Model 1		Model 2		Model 3	
		HR	95% CI	HR	95% CI	HR	95% CI
Never	Never	1	(reference)	1	(reference)	1	(reference)
Ever	Never	2.92	(1.59‐5.36)	2.77	(1.52‐5.06)	2.96	(1.75‐4.99)
Never	Ever	2.73	(1.78‐4.18)	2.76	(1.81‐4.19)	2.79	(1.93‐4.04)
Ever	Ever	8.86	(4.82‐16.30)	8.32	(4.56‐15.18)	8.54	(4.90‐14.87)
	Multiplicative interaction	1.11	(0.74‐1.69)	1.09	(0.73‐1.63)	1.03	(0.76‐1.41)
	RERI	4.21	(2.26‐8.13)	3.79	(2.04‐7.28)	3.79	(2.00‐7.25)
	AP	0.48	(0.40‐0.54)	0.46	(0.38‐0.52)	0.44	(0.37‐0.51)
	Synergy	2.16	(1.83‐2.77)	2.07	(1.77‐2.65)	2.01	(1.75‐2.36)

Note

Model 1: Adjusted for age and area

Model 2: Adjusted for age, area, body mass index, vegetables and fruit intake

Model 3: Adjusted for age, area, body mass index, vegetables and fruit intake. Esophageal cancer arising within 2 years of the start of follow‐up was excluded.

Abbreviations: AP, attributable proportion; CI, confidence interval; HR, hazard ratio; RERI, relative excess risk due to interaction.

Cumulative smoking and amount of alcohol drinking and their interaction for the risk of esophageal cancer is shown in Table 4. Compared to people who did not smoke and drank less than 23 g of alcohol a day, those who drank 46 g or more had an HR of 5.29 (2.90‐9.65) and those with more than 40 pack‐years had an HR of 4.80 (2.97‐7.77) in the age‐ and area‐adjusted model (model 1). These HRs were increased by alcohol consumption in each cumulative smoking level. All interactions between alcohol drinking levels and cumulative smoking with multiplicative evaluation were negative in direction and lacked statistical significance. On the other hand, additively evaluated interactions were positive for risk. In particular, the additive interaction among those with a combination of more than 40 pack‐years of smoking and consumption of 46 g or more of alcohol a day was significantly positive, with an RERI of 8.47 (2.20‐16.16), synergy of 2.05 (1.20‐3.43), and AP of 0.48 (0.16‐0.67). Similar HRs, multiplicative interactions and additive interactions were seen in model 2 (multivariate‐adjusted model) and model 3 (exclusion of early cases).

**Table 4:** Cumulative cigarette smoking, amount of alcohol drinking, and their interaction for the risk of esophageal cancer

Pack‐years	Amount of alcohol drinking (g/day)	HR	95% CI	Multiplicative interaction	RERI	AP	Synergy
Model 1							
0	<23	1	(reference)				
0	≥23, <46	3.43	(1.73‐6.79)				
0	≥46	5.29	(2.90‐9.65)				
≤40	<23	2.75	(1.57‐4.80)				
≤40	≥23, <46	7.81	(4.60‐13.24)	0.83 (0.38‐1.80)	2.63 (−0.36‐5.94)	0.34 (−0.05‐0.58)	1.63 (0.94‐3.05)
≤40	≥46	10.92	(6.50‐18.35)	0.75 (0.35‐1.60)	3.88 (−1.22‐9.75)	0.36 (−0.14‐0.62)	1.64 (0.86‐3.04)
>40	<23	4.80	(2.97‐7.77)				
>40	≥23, <46	12.96	(6.16‐27.26)	0.79 (0.30‐ 2.04)	5.73 (−1.25‐18.58)	0.44 (−0.17‐0.73)	1.92 (0.84‐4.25)
>40	≥46	17.56	(11.45‐26.92)	0.69 (0.32‐ 1.48)	8.47 (2.20‐16.16)	0.48 (0.16‐0.67)	2.05 (1.20‐3.43)
Model 2							
0	<23	1	(reference)				
0	≥23, <46	3.71	(1.86‐7.37)				
0	≥46	5.56	(2.99‐10.33)				
≤40	<23	2.66	(1.55‐4.56)				
≤40	≥23, <46	8.02	(4.78‐13.46)	0.78 (0.36‐1.68)	2.30 (−0.84‐5.49)	0.30 (−0.12‐0.56)	1.53 (0.88‐2.84)
≤40	≥46	10.35	(6.25‐17.13)	0.70 (0.33‐1.47)	3.13 (−2.03‐8.52)	0.30 (0.24‐0.59)	1.50 (0.78‐2.78)
>40	<23	4.84	(3.03‐7.72)				
>40	≥23, <46	12.58	(6.12‐25.86)	0.70 (0.28‐ 1.73)	5.03 (−1.47‐16.53)	0.40 (−0.20‐0.69)	1.77 (0.81‐3.70)
>40	≥46	16.85	(11.07‐25.66)	0.63 (0.30‐ 1.32)	7.46 (1.41‐14.52)	0.44 (0.10‐0.64)	1.89 (1.12‐3.09)
Model 3							
0	<23	1	(reference)				
0	≥23, <46	3.90	(1.84‐8.27)				
0	≥46	5.62	(2.94‐10.76)				
≤40	<23	2.77	(1.53‐5.01)				
≤40	≥23, <46	8.08	(4.61‐14.15)	0.75 (0.32‐ 1.72)	2.40 (−1.13‐5.84)	0.30 (−0.14‐0.57)	1.51 (0.86‐2.97)
≤40	≥46	10.53	(6.04‐18.38)	0.68 (0.32‐ 1.42)	3.14 (−1.70‐8.48)	0.30 (−0.19‐0.57)	1.49 (0.82‐2.64)
>40	<23	5.16	(3.14‐8.50)				
>40	≥23, <46	13.65	(6.66‐27.99)	0.68 (0.27‐ 1.68)	5.58 (−1.16‐17.43)	0.41 (−0.14‐0.69)	1.79 (0.86‐3.60)
>40	≥46	17.51	(11.04‐27.77)	0.60 (0.29‐ 1.26)	7.72 (1.71‐15.29)	0.44(0.12‐ 0.63)	1.88 (1.15‐2.99)

Note

Model 1; Adjusted for age and area.

Model 2; Adjusted for age, area, body mass index, vegetables and fruit intake.

Model 3; Adjusted for age, area, body mass index, vegetables and fruit intake. Esophageal cancer arising within 2 years of the start of follow‐up was excluded.

Abbreviations: AP, attributable proportion; CI, confidence interval; HR, hazard ratio; RERI, relative excess risk due to interaction.

The PAF of esophageal cancer incidence from cigarette smoking and alcohol drinking was estimated (Table 5). PAF from ever smoking, ever drinking, and the combination of both ever smoking and ever drinking was 55.4%, 61.2%, and 81.4%, respectively. Similarly, PAF from cumulative smoking, amount of alcohol drinking, and their combination was 49.7%, 59.5%, and 84.0%, respectively.

**Table 5:** Population attributable fraction of cigarette smoking and alcohol drinking for esophageal cancer incidence

	Cigarette smoking only	Alcohol drinking only	Cigarette smoking and alcohol
PAF1	0.554	0.612	0.814
PAF2	0.497	0.595	0.840

Note

PAF1 was calculated using person‐years and HRs estimated by cigarette smoking status and alcohol drinking status.

PAF2 was calculated using person‐years and HRs estimated by cumulative smoking (pack‐years) and amount of alcohol drinking status.

HRs were adjusted for age, area, body mass index, vegetables and fruit intake.

Abbreviation: PAF; population attributable fraction

## 4 DISCUSSION

We conducted a pooled analysis of eight large population‐based cohort studies to quantitatively estimate esophageal cancer risk of cigarette smoking and alcohol consumption among Japanese males. To our knowledge, this pooled study represents the largest evaluation of the magnitude of the impact of cigarette smoking and alcohol drinking for esophageal cancer. In addition, it is the first pooled analysis of population‐based cohort studies to evaluate the interaction of cigarette smoking and alcohol drinking in both multiplicative and additive scales.

Cigarette smoking alone was associated with a 2.77 times’ higher risk of esophageal cancer in this study. Our previous meta‐analysis of published articles focusing on Japanese populations showed a consistent summary estimate of 3.01.^50^ In addition, pack‐years showed clear dose‐response relationships with esophageal cancer risk. Likewise, alcohol drinking alone was associated with a 2.76 times’ higher risk of esophageal cancer, which was consistent with our previous meta‐analysis.^51^ A clear dose‐response relationship was shown between the amount of alcohol drinking and esophageal cancer risk. Esophageal cancer control might legitimately take account of not only the significance of risk by cigarette smoking and alcohol drinking, but also the magnitude of these risks.

This study assessed the interaction between cigarette smoking and alcohol drinking. When the interaction was assessed multiplicatively, the interaction in ever smoking and ever drinking was non‐significant and slightly positive, whereas the interaction in smoking of more than 40 pack‐years and drinking 46 or more grams of ethanol a day was non‐significant and negative. Therefore, in this study, we did not observe any evidence of multiplicative interaction. In other words, the magnitude of combination effect of smoking and drinking by dichotomous and trichotomous categorization was compatible with what we expected from multiplicative model without multiplicative interaction. In contrast, when we evaluate those interactions in additive scale, the interactions were consistently positive and statistically significant. In addition, magnitude of interaction in additive scale showed dose‐response relationship with cigarette smoking and alcohol drinking. This consistency, dose‐response relationship, and biological plausibility support the idea that cigarette smoking and alcohol drinking interacts in an additive way.

Additive interaction has an interpretation in terms of the presence of biological interaction between the factors. Various gene alterations for esophageal cancer carcinogenesis were reported. Recent study suggested that distributions of gene mutations in physiologically normal epithelia and esophageal squamous cell carcinoma were different.^52^ Thus, accumulation of non‐specific gene alteration in normal esophageal mucosa and esophageal cancer‐specific gene alteration might be necessary for esophageal carcinogenesis. When multistage carcinogenesis theory was assumed, accumulation of non‐specific gene alteration might occur as first stage, then cancer‐specific gene alteration as second stage might be required in esophageal carcinogenesis. Both cigarette smoking and alcohol drinking might mainly affect the first stage because of the additivity of their interaction and long duration of their exposure. Furthermore, cigarette smoking and alcohol drinking were associated with cancer‐specific gene alteration.^52^, ^53^, ^54^ The evidence might support the significant positive interaction in additive scale because both cigarette smoking and alcohol drinking might partly play a role in the second stage.

Results for PAF suggested that cigarette smoking and alcohol drinking cause more than 80% of esophageal cancer. Furthermore, smoking only or alcohol drinking only might cause around 50% and 60% of esophageal cancer, respectively. Simple summation of PAF from smoking and drinking was more than 100% because of the interaction between cigarette smoking and alcohol drinking. It just means that in some individuals, the presence of both factors is required for the cancer to occur.^55^ As such, this is a quantity of interest to public health practitioners because acting on only one risk factor can not only prevent cases associated with exposure to this factor alone but also cases that need both exposures to happen. This also finds a practical translation in PAF estimations: in the absence of additive interaction, the PAF for a modification of exposure to both factors is equal to the sum of the PAFs for the modification of exposure to each factor separately whereas it is less than this sum in case of additive interaction. Thus, both quitting smoking and abstaining from alcohol would be the best way to reduce esophageal cancer incidence. However, either tobacco control or restriction of alcohol would likely provide an adequate degree of impact and might therefore be an option. Indeed, the impact of intervention might be an important point in planning public health policy to achieve esophageal cancer control with fewer costs.

The study has several strengths. First, it estimated interactions by both multiplicative and additive scales. Second, it pooled eight large population‐based cohort studies in Japan. The magnitude of risks and interactions would therefore be valid and applicable to Japanese males. Several limitations should also be mentioned. First, female subjects were excluded from the study. Age‐adjusted esophageal cancer incidence rate per 100,000 Japanese males and females in 2009 was 17.1 and 2.8, respectively.^56^ Moreover, the prevalence of smoking and habitual drinking among females (9.0% and 9.3%, respectively) were much lower than those among males (28.2% and 42.9%, respectively).^57^ Accordingly, any finding that the magnitude of relative risks and interaction in females were similar to those in males would suggest that cigarette smoking, alcohol drinking and their interaction might have less influence on esophageal cancer than in the present study.

In conclusion, we confirmed that cigarette smoking and alcohol drinking were risk factors for esophageal cancer in Japanese males. A significant positive additive interaction between cigarette smoking and alcohol drinking was found, although significant interaction on a multiplicative scale was not observed. PAF of cigarette smoking, alcohol drinking and their combination suggest that either quitting smoking or drinking alone might make a major contribution to esophageal cancer prevention.

## Data Availability

The datasets used in the manuscript are not publicly available. A collaboration with each cohort study groups would be required to access the datasets.
