# Supplementary material for: Revisit of an unanswered question by pooled analysis of eight cohort studies in Japan: Does cigarette smoking and alcohol drinking have interaction for the risk of esophageal cancer?
Source: Cancer Med. 2019 Sep 1;8(14):6414–25. doi: 10.1002/cam4.2514 (PMC6797581; doi:10.1002/cam4.2514)
Supplement: Supplementary file 1 [file CAM4-8-6414-s001.docx]

| Supplemental Table 1. Details of the Study Method in Each Study | | |  |
| --- | --- | --- | --- |
| Study | Variables used in data linkage | Censoring date | Methods to obtain information on cancer incidence |
| JPHC-I | First and last names, sex, date of birth, and address | Date of death, date of emigration, or date of last follow-up, whichever comes first. | Local cancer registries or direct access to major local hospitals |
| JPHC-II | First and last names, sex, date of birth, and address | Date of death, date of emigration, or date of last follow-up, whichever comes first. | Local cancer registries or direct access to major local hospitals |
| JACC | First and last names, sex, date of birth, and address | Date of death or date of emigration | Local cancer registries or direct access to major local hospitals |
| MIYAGI | First and last names, sex, date of birth, date of death, and address | Date of death or date of emigration | Local cancer registry |
| OHSAKI | First and last names, sex, date of birth, and address | Date of death or date of emigration | Local cancer registry |
| 3-pref MIYAGI | First and last names, sex, date of birth, date of death, and address | Date of death or date of emigration | Local cancer registry |
| 3-pref AICHI | First and last names, sex, date of birth, and address | Date of emigration | Local cancer registry |
| TAKAYAMA | First and last names, sex, date of birth, and address | Date of death or date of emigration | Local cancer registries or direct access to major local hospitals |
| JPHC, Japan Public Health Center-based Prospective Study; JACC, The Japan Collaborative Cohort Study; MIYAGI, The Miyagi Cohort Study; OHSAKI, Ohsaki Cohort Study; 3-pref MIYAGI, The Three Prefecture Cohort Miyagi; 3-pref AICHI, The Three Prefecture Cohort Aichi; TAKAYAMA, The Takayama Study | | | |
