# Supplementary material for: Revisit of an unanswered question by pooled analysis of eight cohort studies in Japan: Does cigarette smoking and alcohol drinking have interaction for the risk of esophageal cancer?
Source: Cancer Med. 2019 Sep 1;8(14):6414–25. doi: 10.1002/cam4.2514 (PMC6797581; doi:10.1002/cam4.2514)
Supplement: Supplementary file 2 [file CAM4-8-6414-s002.docx]

| Table S2. Distribution of cigarette smokers and alcohol drinkers | | | | | |  |  |
| --- | --- | --- | --- | --- | --- | --- | --- |
|  |  | Subjects | (%) | Person-years | (%) | Cases | (%) |
| Cigarette smoking status | Alcohol drinking status |  |  |  |  |  |  |
| Never | Never | 15,534 | (10.06) | 181,041 | (9.21) | 30 | (3.29) |
| Ever | Never | 17,725 | (11.48) | 223,074 | (11.35) | 72 | (7.90) |
| Never | Ever | 45,282 | (29.33) | 581,217 | (29.58) | 181 | (19.87) |
| Ever | Ever | 75,828 | (49.12) | 979,850 | (49.86) | 628 | (68.94) |
| Total |  | 154,369 |  | 1,965,182 |  | 911 |  |
|  |  |  |  |  |  |  |  |
| Pack-years | Amount of alcohol drinking (g/day) |  |  |  |  |  |  |
| 0 | <23 | 17,992 | (18.71) | 217,374 | (17.93) | 37 | (6.15) |
| 0 | >23, <46 | 5074 | (5.28) | 63,567 | (5.24) | 21 | (3.49) |
| 0 | >46 | 9112 | (9.47) | 118,579 | (9.78) | 61 | (10.13) |
| <40 | <23 | 19,332 | (20.10) | 243,839 | (20.11) | 52 | (8.64) |
| <40 | >23, <46 | 8347 | (8.68) | 107,613 | (8.88) | 53 | (8.80) |
| <40 | >46 | 17,323 | (18.01) | 230,425 | (19.01) | 151 | (25.08) |
| >40 | <23 | 8128 | (8.45) | 97,998 | (8.08) | 52 | (8.64) |
| >40 | >23, <46 | 2938 | (3.05) | 35,618 | (2.94) | 42 | (6.98) |
| >40 | >46 | 7937 | (8.25) | 97,397 | (8.03) | 133 | (22.09) |
| Total |  | 96,183 |  | 1,212,411 |  | 602 |  |
